# Together, alongside or against each other? A qualitative content analysis on the intersystem sensitivity of coaches and physiotherapists in German elite sport support teams

**DOI:** 10.3389/fsoc.2026.1870432

**Published:** 2026-08-19

**Authors:** Annika Steinmann, David Jaitner, Swen Koerner

**Affiliations:** 1 Institute of Pedagogy and Philosophy, German Sport University Cologne, Cologne, Germany; 2 Department of Training Pedagogy and Martial Research, German Sport University Cologne, Cologne, Germany

**Keywords:** coaches, elite sport, intersystemic sensitivity, physiotherapists, professionalisation

## Abstract

This study examines the systemic dynamic of collaboration and the role of intersystem sensitivity between coaches and physiotherapists in German elite sport support teams. Based on a heuristic that integrates theories of society, conflict, and professionalisation within the framework of Niklas Luhmann’s sociological systems theory, the study employs qualitative content analysis of expert interviews with representatives of both professional groups (*N* = 45). The analysis identifies key themes, conflict potentials and avenues for optimisation in intersystem interaction. The findings suggest that effective collaboration in elite sport support teams depends substantially on the ability to understand and respect the professional logics and goals of other support team members. More specifically, the results indicate that promoting intersystem sensitivity and targeted professionalisation processes is necessary to reduce professional conflicts and optimise the joint care of athletes. The study provides valuable insights into the complexity of elite sport support team dynamics and underscores the importance of a collaborative relationship between coaches and physiotherapists for sporting success and the health of elite athletes.

## 1 Introduction

Modern society constitutes a complex and contingent social formation. Approaches to social problems must therefore generate sophisticated and differentiated solutions. Yet such solutions can never be regarded as fixed in any absolute sense. Rather, they remain contingent and open to alternatives (Luhmann, 1984). Within occupational contexts, professionalism has emerged as a distinct form of social action aimed at coping with this contingent societal complexity at a certain level of quality. Social actors working in “occupations with a professional character” (Dietrich et al., 1990, p. 27) possess licensed expertise in dealing with specified societal problem fields and support their clients in managing everyday situations effectively (Evetts, 2003).

As a specific form of qualified employment, professionalism is tied both to the personal prerequisites of professionals and the structural conditions of occupational work. In practical terms, this presupposes professional action grounded in substantive, communicative, and structural competencies. Beyond the structural competencies required to act professionally within specific institutional and organisational contexts (Käch and Neuhaus, 2018), contemporary workplaces require competent engagement with professionals whose work is shaped by different societal function systems (e.g., law, politics, health, religion). In functionally differentiated societies, these function systems shape interactions and organisations through distinct systemic logics (Luhmann, 1984). Accordingly, occupational settings frequently bring together actors whose orientations are informed by different systemic references. Under such conditions, systemic collaboration and intersystem sensitivity become crucial for minimising structural “conflict potentials” (Luhmann, 1976) and for enabling the professional processing of selected societal reference problems.^1^

Elite sport support teams constitute a paradigmatic example of such intersystemic occupational contexts. Elite athletes are embedded in multi-professional social configurations (Reid et al., 2004) in which incumbents of performance roles from different societal function systems collaboratively address specialised tasks (Collins et al., 2013). In the everyday practice of elite sport support teams, actors associated in particular with the sport system and the health system play a central role in athletes’ lives (Steinmann et al., 2020). This relevance generates multiple points of contact between sport and health, for example with regard to treatment duration, return-to-sport decisions, and the willingness of athletes to compete while injured. Systemic collaboration and intersystem sensitivity therefore appear as key features of professionalism among coaches and physiotherapists in elite sport support teams.

While a growing body of research on elite sport support teams has analysed interactional dynamics and organisational coordination (e.g., Charmant et al., 2021; Malcolm, 2006; Nixon, 1994; Waddington and Roderick, 2002) as well as normative dilemmas between sport and health professionals (e.g., Collins et al., 1999, 2013; Theberge, 2007; Malcolm and Scott, 2014), approaches that conceptualise these issues at the level of society have remained largely absent. As a result, the systemic conditions and interdependencies shaping intersystem practices in elite sport support teams have so far eluded adequate empirical capture. Our study addresses this gap. Drawing on sociological systems theory, we aim to reconstruct empirically the systemic dynamics of collaboration and intersystem sensitivities of two actors who are particularly important in athletes’ support environments: coaches and physiotherapists. Based on expert interviews with representatives of both groups from various Olympic sports in Germany, we employ qualitative content analysis to examine the intersystemic expectation structures and meaning-processing patterns through which they coordinate and interpret their interactions across functional system boundaries.

## 2 Theoretical framework and research context

### 2.1 Systemic collaboration and intersystem sensitivity: a systems-theoretical heuristic

Our analysis draws on Niklas Luhmann’s sociological systems theory (Luhmann, 1984). As a general sociological framework, this approach is grounded in the assumption that society is constituted by social systems and that communication, rather than action or the individual, forms the basic element of the social. Social systems are therefore understood as systems of communication that reproduce themselves through communication alone. In Luhmann’s terms, social systems are autopoietic and operationally closed. They do not share operations with biological systems (life) or psychic systems (consciousness), but can reproduce themselves only through their own specific operation, namely communication.

Communication itself emerges through the synthesis of three selections: information, utterance, and understanding. Ego selects a piece of information and a form in which this information is uttered. Alter, in turn, must distinguish between information and utterance and process this distinction as meaningful. Understanding does not imply agreement or successful reception in any substantive sense. Rather, it denotes the recognition of an utterance as communicative and thereby establishes the possibility of further communication. In this way, communication does not consist in the transmission of meaning from one individual to another, but in specifically social operation that recursively connects to further communication (Luhmann, 1984, pp. 193ff.).

The architecture of Luhmann’s theory combines a theory of observation with a theory of society. The theory of observation starts from a difference-theoretical and constructivist premise (Luhmann, 1988). Following Spencer-Brown (1969), observation is understood as the “indication of something in the context of a […] distinction from something else” (Luhmann, 1984, p. 596). This conceptual move is crucial because it implies that every observation is selective and contingent: To observe something always means to distinguish from something else. As a theory of society, Luhmann’s approach explains why such observations are not structured randomly, but according to historically specific forms of social differentiation. The defining structural principle of modern society is functional differentiation. Functional differentiation means that modern society is not organized hierarchically around a single center, but is constituted primarily by a plurality of function systems that are distinct, non-interchangeable, and specialised in relation to different societal reference problems. Each function system reproduces its communications according to its own binary code and thereby establishes a specific social perspective on the world. These codes operate as guiding distinctions that reduce complexity by making communication connectable in system-specific ways. Thus, function systems do not merely coexist. They structure social reality through different and often competing logics of relevance, evaluation and problem processing (Luhmann, 1984).^2^

On this basis, we conceptualize *systemic collaboration* not as a cooperation between systems in a literal sense, but as a specific form of coordinated interaction among organisationally embedded actors whose communication is shaped by different functional logics. Intersystemic constellations arise wherever multiple system references become relevant within the same interactional context. This is particularly important in modern society because interactions are never socially neutral. They unfold against the background of a fully inclusive society (Barth, 2023) in which different function systems can become simultaneously relevant to the same situation. Under such conditions, the communication of those involved may be informed by divergent logics and criteria of appropriateness.

The societal, organisational, and interactional dimensions should not be understood as competing explanations, but as analytically distinct forms of social system formation (Luhmann, 1975). Coaches and physiotherapists encounter one another in concrete interactions, while these interactions are commonly situated within organisational contexts such as clubs, federations, and training centres. Such organisations are multireferential: Through their decisions, programmes, role structures and communication channels, they can selectively process references to several societal function systems rather than merely reproducing a single functional logic (Lieckweg and Wehrsig, 2001). Organisations therefore mediate the encounter between sport- and health-related expectations by allocating responsibilities and resources, establishing decision-making authority, and specifying legitimate forms of communication. The societal perspective remains necessary because it identifies the differentiated functional references that cannot be derived from organisational structures alone. The organisational perspective explains how these references are selectively prioritised and translated into decision premises, while the interactional perspective shows how the resulting expectations are enacted and negotiated in concrete situations. Our heuristic primarily foregrounds the societal dimension, while treating organisations and interactions as the social contexts in which intersystemic differences become practically observable and consequential.

Where multiple functional logics are present within one social situation, different constellations are possible. First, they may remain largely indifferent to one another and operate side by side without substantial coordination. In this case, the involved perspectives coexist, but do not become meaningfully integrated. Second, they may become linked in a collaborative way, such that substantial solutions emerge which take more than one functional logic into account. Third, these same intersections may take an antagonistic form, namely when different system references are mobilised against one another and reciprocal expectations are rejected rather than coordinated. The relationship between different functional logics is therefore not fixed but situationally contingent and open to different forms of stabilisation.

Conflict is of particular importance in this context. In systems-theoretical terms, conflicts are not external disruptions of communication, but specific forms of communication in their own right (Luhmann, 1984, Ch. 9). They arise when communication is negated and this negation is answered by a further negation, that is, “when a no is answered with a counter-no” (Luhmann, 1993, p. 565). The basis of conflict lies in unfulfilled expectations. Luhmann (1976) refers to the structural possibility of such expectation divergences as “conflict potentials.” These potentials become socially consequential when expectations are articulated and met with unacceptable counter-communication (e.g., genuine disagreement with previous communication, strategic contradiction). Conflict thus marks a specific mode in which intersystemic relations can become stabilised, namely through contradiction rather than accommodation.

Against this background, a key factor shaping whether intersystemic constellations remain indifferent, become collaborative, or escalate into antagonism lies in the structural competences of the actors involved. Within these competences, *intersystem sensitivity* is of central importance. We understand intersystem sensitivity as the capacity to recognise which functional logics are relevant in a given situation, to understand that the actions of others are oriented towards different but socially legitimate system references, and to incorporate this awareness reflexively into one’s own communicative practice. In this sense, intersystem sensitivity does not imply dissolving systemic difference. Rather, it refers to a practically relevant competence for dealing with such differences in a way that reduces conflict potentials and enables coordinated interaction across functional boundaries.

### 2.2 Systemic collaboration and intersystem sensitivity as a key feature of professionalism in functionally differentiated work contexts

To characterise systemic collaboration and intersystemic sensitivity as features of professionalism, we situate our analysis within differentiation theoretical approaches to professional action. Differentiation-theoretical models of professional action are rooted in the tradition of the structural-functionalist approach to professions (Parsons, 1964). They emphasise, however, the comparison of different societal subsystems and the particularities of professionalised social systems (e.g., Kurtz, 2000; Stichweh, 1992). The starting point of these approaches is the theorem of functional differentiation. In this context, especially in social systems oriented toward targeted influence of the included population, professional systems emerge in which certain occupational groups “interpret, manage, and process” (Kurtz, 2005, p. 245) key issues of the personal environments of these social systems in a representative manner.

The professional actors thereby assume occupational performance roles that attend to the guiding distinctions of a social system and align their actions primarily with the ‘positive’ value of the system-specific coding. The involvement of relevant population members occurs in a manner complementary to these occupational performance roles and is characterised by asymmetrical system expertise between the professional role and the clients (Stichweh, 1992).^3^ Professional action is distinguished by its capacity to apply “socially approved solutions” (Pfadenhauer, 2005, p. 11) to specific societal problems in a professionally competent and individually appropriate manner. The proximity to complex individual problems means that professional actors are continually confronted with concrete cases, which must be translated into situationally appropriate decisions and forms of interaction (Pfadenhauer, 2005). The success of “these ‘operations’ of people processing” (Luhmann, 1982, p. 192) is thus inevitably tied to the autopoiesis of the involved systems, the fundamental uncertainty of communication among those present, and the specific characteristics of social situations, such as time pressure, incomplete information, or urgent decision-making requirements. In this sense, professional action is marked by a “technology deficit” (Luhmann and Schorr, 1979), requiring context-sensitive rather than formulaic solutions.

Professional action therefore demands a type of personnel capable of understanding individual problems *in situ* in light of established societal values, such as legal validity, educational success, health restoration, or sporting performance, developing appropriate solutions and implementing them. For this to be possible, professional role bearers require institutionally specialised educational pathways, including vocational training, higher education, and certified continuing education, that provide certified access to scientifically grounded bodies of specialised knowledge and enable actors to relate reflexively to this expertise and their professional practice (Kurtz, 2005). This necessitates comprehensive substantive, communicative, and structural competencies. Substantive competencies refer to domain-specific expertise. Communicative competencies concern the capacity to translate expertise into interactional forms that are understandable and practically relevant for others. Structural competencies pertain to social structures, i.e., rule-governed social facts that frame action in work contexts and constrain the contingency of social action. Beyond institutional (e.g., laws, professional codes, public expectations) and organisational (e.g., corporate structures, responsibilities, procedural regulations) structural features (Käch and Neuhaus, 2018), professional action in functionally differentiated work contexts is fundamentally shaped by the functional logics of the social systems involved in concrete professional situations (Stichweh, 1992).

Given that contemporary multiprofessional work environments continually entail points of contact between different social systems (Vollmer, 2016), systemic collaboration and intersystem sensitivity represent core markers of professionalism. They serve to minimise structurally induced “conflict potentials” (Luhmann, 1976) and to mediate successfully between the ‘positive’ values of systems and the included clients (Luhmann, 1982). Professional role-bearers who act with intersystem sensitivity are able to identify relevant societal guiding distinctions, recognise the system references that shape the expectations of other actors, and incorporate these differences into their own communicative practice. This does not mean that they abandon the logic of their own profession or dissolve functional differentiation into consensus. Rather, it means that they can make systemic differences practically manageable. In this sense, intersystem sensitivity is a structural competence that enables professionals to deal reflexively with divergent expectations, to anticipate potential contradictions, and to contribute to forms of collaboration that are not merely personal or *ad hoc*, but professionally and structurally stabilised.

### 2.3 Intersystemic professionalisation of coaches and physiotherapists in elite sport support teams

Modern elite sport consolidates societal communications that are meaningfully oriented towards achieving victories and avoiding defeats in athletic competition (Körner, 2013). The demand to consistently produce superior athletic performance drives an increasing differentiation of personnel, resources, expertise, and specialised training infrastructures. To gain and maintain competitive advantage, elite athletes are supported by multiprofessional support teams (Reid et al., 2004). The professional actions of these supporting actors, including coaching staff, medical departments, psychological support, administrative staff, management, scientists, and nutritional advisors, unfold primarily at the intersection of different societal subsystems. Coaches and physiotherapists are central performance roles within these support teams, because their everyday work repeatedly brings the logic of sport into contact with the logic of health.

Elite sport coaches operate primarily in relation to the victory/defeat code (Schimank, 1988) and bear primary responsibility for developing peak athletic performance, especially through the planning, design, implementation, and evaluation of training and competition situations, as well as coordinating the support team environment. Coaches exist in a complementary relationship with the athletes they supervise and are heavily dependent on the athletes’ sporting success. Characteristic features of this performance role include time-limited employment, limited job security, high personnel turnover and, in some cases, part-time engagement (Cachay, 1995). These structural vulnerabilities of the performance role have increasingly activated professionalisation needs in recent years and indicate signs of a professionalisation process that contributes to stabilising the occupational position (Digel et al., 2010; Hartmann-Tews, 1999; Lüsebrink, 2001; Patsantáras, 1994).

Physiotherapists in elite sport contribute to the prevention, treatment, and rehabilitation of functional disorders of the musculoskeletal system, while aligning their professional practice with the health system’s distinction between illness and health (Luhmann, 1990). As a healthcare support profession, the formal subordination to medical authority characterizes the physiotherapy performance role. Without additional qualifications (e.g., sectoral medical practice in Germany), physiotherapists are not legally authorized to practice independently and must follow medical prescriptions (Hüter-Becker, 2010; Scott and Malcolm, 2015). In elite sport, physiotherapists, through their typical hands-on approach in individual treatments, temporal flexibility, availability, treatment duration, and irreplaceable expertise, become significant reference and trust figures for athletes (Scott and Malcolm, 2015; Malcolm, 2016, Ch. 8; Charmant et al., 2021).

However, within the elite sport “culture of risk” (Nixon, 1992, 1994), where pain and health impairments are treated as “routine and uneventful” (Curry and Strauss, 1994, p. 195), the physiotherapy performance role and its “culture of precaution” (Safai, 2003) is regularly embedded in situations involving both elite sport and health logics, e.g., treatment duration, return-to-play decisions, playing hurt practices, and presenteeism (Bolling et al., 2020; Malcolm, 2006; Mayer and Thiel, 2011; Mayer et al., 2020; Reussner and John, 2025; Theberge, 2007). Coaches often appear to have insufficient knowledge about the physiotherapy profession and medical contexts, frequently interpreting the physiotherapy performance role uncritically as support for athletic performance (Steinmann et al., 2020). This can generate expectations that physiotherapists provide rapid treatment effects, continuous information and sport-specific availability. Physiotherapists are positioned between professional ethical obligations to the athlete as a patient and organisational loyalty to the club and coach. Waddington and Roderick (2002) highlight managing confidential information as a key challenge. In this respect, no unified professional orientation appears to exist across all contexts. Some physiotherapists primarily see themselves as club employees, others as confidants to athletes. The potential misuse of medical data and the pressure to disclose information for sporting purposes, possibly against confidentiality obligations, pose ethical and professional autonomy challenges (Waddington and Roderick, 2002; Malcolm and Scott, 2014). Consequently, there are ongoing efforts to professionalize the field and enhance occupational autonomy in elite sport physiotherapy (Groll et al., 2005; Klemme et al., 2007; Scott and Malcolm, 2015; Steinmann, 2019).

The collaboration between coaches and physiotherapists is therefore not merely an interprofessional coordination problem. It is an intersystemic constellation in which different definitions of success, different temporal horizons, and different professional obligations meet. While reciprocal interpretive schemes and functional expectations among actors within the sports system do not generate macrostructural contradictions, intersystemic contacts between actors of the sports and health systems reveal differentiation possibilities, underscoring the importance of intersystemically sensitive professional action (Theberge, 2007). They show that the same situation can be observed and evaluated differently depending on whether it is framed through the logic of elite sport or through the logic of health.

The conjoint activity of coaches and physiotherapists in elite sport is thus doubly exposed to “conflict potentials” (Luhmann, 1976) that can impede successful intersystemic cooperation between these two groups. On the one hand, the ostensibly neutral functions of the social systems are differently valued in practice within elite sport support teams, as the logic of sport and the dominant position of coaches, e.g., “coach as king” (Malcolm and Sheard, 2002, p. 165), regularly override the health system’s preventive culture and professional physiotherapeutic practice, for instance, when coaches expect athletes to perform despite injuries (Anderson and Jackson, 2013; Malcolm, 2016, Ch. 8; Collins et al., 1999; Steinmann et al., 2020). On the other hand, the approach of empowering both professional roles through professionalisation processes provides a rationale for intersystem sensitivity. As professionalism increases, so do the complexity and contingency of the professional activities of the two roles. While this increase is interpreted by the profession as intensified professional identity and expanded decision-making capacity, the broader degrees of freedom in professional environments simultaneously reduce expectation certainty. Consequently, bilateral professionalisation necessitates the re-coordination of social relationships under emancipated conditions. In this context, systemic collaboration and intersystem sensitivity emerge as a crucial professional solution, especially when a more symmetrical configuration of action is sought (Willke, 2006, Ch. 2).^4^

## 3 Materials and methods

The present study forms part of a larger research project that aims to systematically analyse the systemic collaboration between coaches and physiotherapists in German elite sport and, on this basis, to develop empirically grounded working materials for intersystem sensitivity (Breucker et al., 2023). The study was approved by the Ethics Committee of the German Sport University Cologne (approval number: 90/2021).

To identify typical reciprocal interpretation schemes and functional expectations between sports and health in elite sport support teams, we conducted expert interviews with coaches and physiotherapists in German elite Olympic sports and analysed them using qualitative content analysis. The qualitative research design allows for theoretically informed reconstructions of professional interpretations, meanings, and expectation structures from the actors’ perspectives, thereby contributing to a differentiated understanding of the targeted social reality (Flick et al., 2010).

The interviewees were sampled purposively (Patton, 2015, pp. 264f.). To be included in the sample, participants had to be actively working in senior-level German elite sport as certified physiotherapists or coaches and had to have at least 1 year of professional experience in this field at the time of the interview. Recruitment was carried out in cooperation with selected elite sport partners: the Trainer Academy of the German Olympic Sports Confederation, the M.Sc. in Sports Physiotherapy at the German Sport University Cologne, the Olympic Training Centre NRW/Rhineland, and the German Swimming Association. The sample comprises 19 coaches (*n_f_* = 3) and 26 physiotherapists (*n_f_* = 12). The coaches have between five and 45 years of experience in elite sport (M = 18.6 ± 11.8 years) and are involved in 12 Olympic sports. They are predominantly employed full-time on fixed-term contracts in either women’s or men’s sport and operate at various levels (e.g., national, state, regional training centres, club level). Eleven coaches have a personal background in elite sport. The physiotherapists have between two and 31 years of professional experience in elite sport (M = 12.1 ± 8.8 years) and contribute perspectives from 28 Olympic sports. Most are employed full-time, work as freelance/self-employed practitioners, or hold permanent positions. Four physiotherapists have personal experience as former elite athletes.

We collected the data between June and September 2021. To protect the health of the interviewees during the COVID-19 pandemic, the interviews were conducted by telephone. All interviews were recorded, transcribed, and anonymised. Transcription followed the transcription procedures proposed by Gläser and Laudel (2010, pp. 193ff.). The interviews lasted between 24:18 and 78:37 min, with an average duration of 47:37 ± 11:36 min. Written informed consent and data protection agreements were obtained from all interviewees.

Drawing on expert interviews, we treated the interviewees as professional experts, thereby enabling the analysis of specialised cultural knowledge and occupational interpretive patterns (Gläser and Laudel, 2010). A semi-structured interview guide was developed for the interviews. During the interviews, the guide served as an orientation framework and was applied flexibly to allow the experts to prioritise topics they considered most relevant. The thematic areas covered in the guide included the following aspects: one’s own role and the roles of others (e.g., typical working days, tasks, responsibilities, and accountability), cooperation between roles (e.g., collaboration and relationships between roles, decision-making authority, potential sources of conflict and actual conflicts, suggestions for improvement), and collaboration with other members of the support staff. In addition to the interviews, a written pre-interview questionnaire and a postscript were used to document contextual information and systematically reflect on the interview process.

The interviews were analysed using structuring qualitative content analysis following Kuckartz and Rädiker (2023). The analysis was conducted with the support of MAXQDA, a software for computer-assisted qualitative and mixed methods data analysis. In a multi-stage process, we developed a coding scheme using a deductive-inductive approach. The coding process was guided by four deductive main categories, which were subsequently differentiated inductively into subcategories (Table 1). For each main and subcategory, working definitions, anchor examples, and, where necessary, coding rules for delimitation were formulated (Supplementary Table 1). To ensure high intercoder reliability and transparent coding guidelines, the category system was consensually validated through a 2-day internal research workshop using 10% of the total interview material. This was followed by coding of the full dataset. The final analysis was additionally validated through a one-day group Delphi procedure (Niederberger and Renn, 2018). In this process, participating coaches (*n* = 5) and physiotherapists (*n* = 3) were confronted with ambiguous or contradictory findings. Through open exchange in moderated discussion rounds, consensus or structured disagreement was reached regarding selected reciprocal intersystemic interpretative patterns and functional expectations.

**Table 1:** Overview of main and subcategories (including codes).

Main category (codes)	Subcategory (codes)
Description of one’s own professional field of action (905)	Professional tasks and qualifications (411)
	Non-professional tasks (15)
	Structural conditions (249)
	Employment status and remuneration (36)
	System conflict (128)
	Relationship with athletes (66)
Description of the other professional field of action (473)	Professional tasks and qualifications (200)
	Non-professional tasks (12)
	Structural conditions (138)
	Employment status and remuneration (39)
	System conflict (33)
	Relationship with athletes (51)
Description of cooperation (710)	Cooperation between coaches and physiotherapists (252)
	Cooperation between physiotherapists, coaches, and third parties (195)
	Structural conditions (60)
	Criticism and conflicts (134)
	Hierarchy (69)
Optimisation approaches (260)	Professional aspects within one’s own field of action (34)
	Professional aspects within the other field of action (22)
	Cooperation (57)
	Structural conditions (147)

## 4 Findings

Our findings reconstruct how coaches and physiotherapists describe their own professional domains, how they describe the other professional domain, and how they interpret the interaction between both domains in everyday elite sport support team practice. To illustrate the findings, selected direct quotes from interviewees of both domains are included. In line with the theoretical framework, the findings are not treated as individual opinions alone, but as empirical indications of role-specific expectation structures and meaning-processing patterns at the intersection of sport and health.

### 4.1 Own professional domains

Regarding their own domain, the *physiotherapists* report being professionally responsible for the physical treatment of athletes, primarily in the areas of regeneration, prevention, rehabilitation, and the restoration or maintenance of training and competition readiness. This includes pain management, optimisation of mobility, and the use of decongestive measures such as lymphatic drainage. To act in a professional manner, continuous professional development is necessary. As highly trusted confidants, they frequently find themselves in situations requiring psychological support for athletes despite not being formally trained for such tasks, and express a recurring need for more professional expertise in this area: “When they are lying on the bench like that and we are alone, you do hear a thing or two, and quite often you are also asked for advice” (#P12). On competition days, their responsibilities shift to immediate care tasks, such as taping, stretching, and massage. Physiotherapists additionally assume assisting tasks in logistical contexts, for example preparing and providing beverages or carrying athletes’ equipment. Physiotherapists are often continuously reachable for their athletes: “They can contact me 24/7″ (#P1). They primarily collaborate with coaches during competitions and training camps. Routine coordination in the day-to-day training context occurs almost exclusively when physiotherapists have treatment rooms or practices located directly on the sports premises, allowing for in-person interactions. Otherwise, exchanges occur on an as-needed basis (e.g., return-to-play decisions). Physiotherapists are mostly employed in private practices or manage such practices. They typically work with elite athletes across multiple sports, genders, and age groups, providing support at competitions or training camps as freelance practitioners, while also treating other patients who are not involved in elite sports: “I am self-employed and run my own practice. I am registered with two professional sports federations, which then contact me when they need my services” (#P2). Only two of the surveyed physiotherapists work exclusively in a single sport, each serving only one (extended) team. Physiotherapy services are generally billed via medical prescriptions or budgets. This does not apply to telephone on-call duties or accompaniment to competitions and training camps, which are performed voluntarily in their free time or compensated with a nominal fee (100–150 EUR/day) that rarely covers actual costs incurred, such as revenue loss in the practice. Physiotherapists utilize all available medical interventions to render athletes ready for competition. Health risks and injuries are sometimes partially accepted during major sporting events: “Even if I’m not supposed to […], these are all adult and fully grown athletes, so I also advise that a painkiller could be taken” (#P1). However, some physiotherapists initiate a time-intensive “targeted problem analysis” of physical complaints and “are not primarily acting as this buddy-physio” (#P21). This points to a tension within the physiotherapy role itself: physiotherapists are expected to restore performance quickly, while also understanding themselves as health professionals responsible for sustainable care. Structurally, the interviewed physiotherapists perceive their limited availability, particularly in less prominent sports due to underfunding, as a major constraint. In training camps, for example, physiotherapists sometimes work “like on an assembly line” (#P3), which undermines the provision of adequate professional care for athletes. As a result, highly qualified and experienced practitioners regularly withdraw from elite sport, as the field is seen as economically unviable and offers limited professional satisfaction.

The *coaches* report that in addition to coordinative and administrative tasks, their main responsibilities involve sport-specific, tactical and technical training, and sometimes conditioning and strength training. They also scout and nominate athletes as well as members of the support team. They lead competition support by preparing their athletes, coaching during competition, and reviewing the competition afterwards. They direct the support team with the goal of achieving the athletes’ Olympic or elite sport success. Coaches are generally not involved in non-professional or assisting tasks. At the highest performance level, coaches are usually supported by assistant coaches. They typically lead two training sessions per day on 5 to 6 days per week, while national coaches train their athletes primarily during camps and competitions. In contrast to physiotherapists, nearly all interviewed coaches are employed full-time, but their appointments are limited in duration. Rarely do coaches have other professional engagements, such as teaching, studying, or employment in customs service. In preparation for major sporting events, such as Olympic Games or World Championships, coaches may accept prolonged injury periods for their athletes. They acknowledge that elite athletic performance can conflict with health, but they also recognise that sustained performance is only achievable with long-term absence of pain or a certain level of health. In the context of major sporting events or at the end of a career, however, all considerations are generally subordinated to the logics of elite sport. For younger athletes and outside major competitions, coaches are more cautious: “Before I burn someone out or totally shred them, I have to ensure they return to training and competition in a sustainable way” (#C10). Coaches consider themselves partly responsible for protecting athletes from themselves, as athletes always want to train and compete despite physical complaints. Their communication with athletes is seen as geared towards reaching consensus, “on equal footing, […] transparent” (#C6).

Taken together, both professional groups describe themselves as indispensable parts of elite sport support teams and as responsible for specific domains of athlete support. Both emphasise high responsibility, high availability, and the practical relevance of their work for athletes. Both are also confronted with the tension between short-term performance expectations and longer-term athlete health. However, their role realities differ markedly. Physiotherapists describe an expanded, sometimes diffuse role that includes therapeutic, emotional, logistical, and availability-related tasks under often precarious structural conditions. Coaches describe a more clearly delimited and hierarchically central role focused on performance development, team coordination, and decision-making authority. This asymmetry forms an important structural background for the interaction between both domains.

### 4.2 Other professional domains

*Physiotherapists* regard sport-specific training as the exclusive responsibility of coaches. This includes the preparation and follow-up of training sessions, error analysis and correction with regard to athletes as well as their continuous monitoring. On competition days, coaches prepare athletes, develop strategies, and give instructions. Coaches are described as the ones “making the corresponding announcements for athletes and the support team. That is entirely within the competence of the coaches. I would never presume to do that” (#P12). This indicates that physiotherapists generally recognise the coaches’ domain-specific authority within the sport system. Some physiotherapists emphasise that coaches support athletes’ dual careers and also undertake organisational tasks on behalf of their athletes, such as coordinating with schools or arranging transportation, so that athletes can perform optimally. Coaches are almost always available on-site because they are employed full-time or directly financed and contracted by the athletes. Alongside physicians and physiotherapists, coaches have an advisory role for athletes and must sometimes make compromises regarding load management and athlete deployment. Among the surveyed physiotherapists, it is undisputed that coaches push for the fastest possible return after injuries and, in important or final competitions, want to field athletes even despite health handicaps. Physiotherapists perceive the relationship between coaches and athletes as age-specifically individualised: there are “buddy-type and helicopter coaches” (#P2), coaches with close personal relationships who assume a fatherly role as well as coaches who do not establish a private level of interaction with their athletes. Compared to physiotherapists, coaches maintain a more distant relationship with their athletes. Athletes sometimes withhold information from coaches to avoid showing weakness, which could, for instance, affect their selection. Coaches sometimes transfer their performance pressure onto the athletes under their supervision.

With regard to the physiotherapists’ domain, *coaches* perceive physical healthcare and, in particular, the wellbeing and “body care” (#C15) of athletes as the primary professional responsibility of physiotherapists. For the coaches, physiotherapists work both preventively to counteract overload and correct muscular imbalances, and rehabilitatively in cases of acute injuries. In the context of competitions, it is the physiotherapists’ task to quickly resolve minor injuries such as bruises or muscle stiffness and to “perk up” athletes (#C5). This indicates a functional interpretation of physiotherapy as an important means of securing training and competition readiness. Most coaches struggle to define these tasks precisely or to identify specific qualifications or training content: “So, they are physio, psychologist, mental coach, hand healer, everything” (#C19). Coaches expect physiotherapists to provide unconditional information regarding the healing processes of injured athletes and to have a very precise understanding of sport-specific loads. Coaches ascribe to physiotherapists the role of a trusted person for athletes: “There is chatting, there is laughing, there is good atmosphere” (#C18). In addition, physiotherapists are partly described as service providers for athletes who ensure good team morale and undertake organisational tasks, such as providing food and drinks, handling transportation, booking hotels. Coaches generally desire even greater availability and personnel continuity from physiotherapists. Clubs and federations, however, are usually unable to provide permanent employment due to financial constraints, so physiotherapists must typically be released for camps and competitions, which is not always straightforward. Coaches recognise that physiotherapists operate at the interface between maintaining health and achieving performance and sometimes engage in exchanges of arguments that lead to compromise solutions: “Training without regard for physical losses” can trigger conflicts, but coaches may also insist on “not needing a physio who wraps the athletes in cotton wool” (#C18). Physical proximity is for coaches the key trigger for a good and personal relationship between physiotherapists and athletes. Some coaches intentionally attempt to gain access to relevant intimate information from athletes via physiotherapists. This expectation points to a sensitive boundary issue: what coaches perceive as useful information for performance steering may conflict with physiotherapists’ professional obligations regarding trust, confidentiality, and athlete-centred care.

Across both perspectives, the other professional domain is recognised as necessary and functionally differentiated. Physiotherapists generally accept the coaches’ responsibility for sport-specific performance development, while coaches recognise physiotherapists as indispensable for health-related support and physical readiness. At the same time, each group observes the other through its own systemic reference. Physiotherapists tend to perceive coaches as performance-driven, risk-accepting, and oriented towards competition. Coaches tend to perceive physiotherapists as responsible for prevention, rehabilitation, and the restoration of functional athlete bodies, but sometimes as too cautious, too academically oriented, or insufficiently adapted to elite sport loads. These reciprocal descriptions already contain the expectation divergences that later become relevant in cooperation.

### 4.3 Interaction between the professional domains

Reflecting on the interaction between coaches and physiotherapists in elite sport support teams, the *physiotherapists* report that there is generally a collegial but personally dependent relationship between the two groups, provided that collaboration exists at all: “[...] but we rarely come together at a table to actually discuss things on a professional level; instead, I often feel put in the role of a car mechanic: fix this!” (#P18). Collaboration with other members of the support team, such as rehabilitation coaches, usually occurs side by side rather than together. Physiotherapists report that, in everyday practice, they sometimes have no contact with the coaches: “There is no collaboration [...] There are no agreements with coaches at all” (#P3). Return-to-sport decisions, load management and competition readiness present the main intra- and interpersonal conflict potential and are frequent occasions for sometimes heated discussions between coaches and physiotherapists. This generally occurs when, often in the context of significant competitions, the health-oriented approach of physiotherapists meets the performance-driven focus of coaches and athletes: “It’s clear that for some unimportant, small-level competitions, one naturally evaluates risks differently than for the Olympic final” (#P2). Physiotherapists therefore do not deny the relevance of performance contexts. Rather, they describe the need to balance health risks differently depending on the situation. This balancing, however, often lacks stable communicative and organisational procedures. In interactions with coaches, it is advantageous to have a strong understanding of the sport being supported, or to have competed at a high level oneself, in order to better contextualise motor-specific demands and training loads. Physiotherapists expect coaches to have a more solid understanding of physiological processes, for instance to avoid disrupting wound healing phases through inappropriate training stimuli. In this context, one physiotherapist reports a coach’s reaction to the communication of a medical assessment in the presence of a jointly supervised athlete: “That’s all too complicated for me, and I also do not understand why it has to be so complicated. I simply want you to function properly” (#P21). At the same time, physiotherapists wish for a broader appreciation that contemporary physiotherapy also involves the observation of movement execution. They therefore seek to be involved and remunerated “on track” (#P21) and to have their professional expertise recognised by coaches beyond “external operations” (#P5) aimed at immediate symptom relief. From their perspective, collaboration would therefore require earlier and more systematic integration into training processes rather than episodic involvement after problems have already occurred.

Following the *coaches’* perspective, interaction between coaches and physiotherapists is characterised by mutual trust, appreciation, and professionalism: “Physiotherapists are usually incredibly pleasant people who are always committed to making things better for others. [...] The staff is incredibly familiar with each other. [...] as professional as necessary and as personal as possible” (#C10). However, many coaches see closer integration as a key avenue for improving professional collaboration: “You actually almost never talk to them” (#C8). An exception to this is training camps. Facilitated by spatial proximity, communication and direct professional contact take place in this setting. Overall, roles seem to be clearly delineated. Physiotherapists attend to the health concerns of jointly managed athletes, while coaches are responsible for all sport-specific aspects. Collaboration with other members of the support team, such as rehabilitation coaches, occurs only on an as-needed basis. Some coaches do not seek contact with physiotherapists, prefer not to integrate physiotherapy into training, and reject observations by physiotherapists during training in order to train undisturbed and without external intervention. However, there are also coaches who report “very, very close collaboration with physiotherapists,” which includes reciprocal exchange (#C19). The majority of coaches wish for physiotherapists to be present on-site: “[...] then they should come to the training facility, to normal training, and see how the athletes actually move” (#C8). At the same time, they criticise the perceived decline in manual skills among physiotherapists and a lack of understanding of elite sport training loads. In their view, this is primarily driven by low remuneration, which results in high staff turnover and a predominance of early-career practitioners: “Every day they spend with us costs them 2,000 Euro in revenue. So they really have to be pretty stupid, in a way” (#C10). From a structural perspective, coaches therefore see potential for improvement in ensuring greater staff continuity and expanding the physiotherapy department: “[…] that physiotherapists actually have enough time at all locations to attend to athletes individually” (#C18). Moreover, the coaches argue that the increasing academisation of physiotherapy is pushing the craft-based aspects of the profession further into the background: “People are now trying to do the whole thing through a university degree. but there are some very poor physiotherapists” (#C1). From the coaches’ perspective, conflicts arise, when physiotherapists “push themselves to the forefront or are not available” (#C18), present themselves as “hobby psychologists” since they “are not psychologists” (#C6), or when “the flow of information is not as it should be” (#C13). These formulations reveal a specific expectation structure: physiotherapists are valued when they support performance readiness and provide useful information, but they are criticised when they appear to exceed the role boundaries defined from the coaches’ perspective. From the perspective of most coaches, physiotherapists have the task of creating the physical prerequisites for athletic performance, producing functional athlete bodies. However, some coaches also consider physiotherapists as part of their team, which they lead: “There is a person responsible for the group [...] and that is me” (#C1). In the event of conflicts, “[...] it is then, let us say, more likely that the physiotherapist changes than that the athlete changes the coach” (#C17), similarly, “we have to terminate the “collaboration” (#C18)”.

Overall, the interaction between coaches and physiotherapists oscillates between working alongside, working together, and working against each other. Alongside constellations emerge where both groups fulfil their respective tasks in parallel, with limited communication and little institutionalised coordination. Together constellations occur where spatial proximity, personal trust, and reciprocal understanding enable case-related exchange and joint problem-solving. Against constellations emerge when health- and performance-related expectations are mobilised against one another, particularly in return-to-sport decisions, load management, competition readiness, information disclosure, and role boundary negotiations. The findings therefore indicate that collaboration is not only a matter of individual willingness or mutual knowledge. It is also a systemic and structural problem. Conflicts arise from divergent definitions of success, weakly institutionalised communication, asymmetric power relations, and unequal resource conditions. Intersystem sensitivity appears to be present in partial and situational forms, especially where actors recognise the relevance of the other domain and acknowledge the need for compromise. However, it is rarely stabilised as an organisationally supported form of professional practice. Instead, systemic collaboration remains largely dependent on personal relationships, local proximity, and *ad hoc* coordination. This makes the cooperation between coaches and physiotherapists vulnerable to breakdown precisely in those situations in which intersystem sensitivity would be most needed.

## 5 Discussion

The present study examined systemic collaboration and intersystem sensitivity between coaches and physiotherapists in German elite sport support teams. Drawing on sociological systems theory (Luhmann, 1984), we analysed how representatives of both professional groups describe their own professional domains, how they observe the other domain, and how they interpret everyday interaction between both domains. The findings show that collaboration between coaches and physiotherapists is shaped by a persistent tension between the performance orientation of elite sport and the health-oriented logic of physiotherapeutic practice. This tension does not simply result from personal misunderstanding, insufficient knowledge or deficient personal communication. Rather, it reflects structurally embedded differences between societal system references, professional role expectations, organisational arrangements and asymmetrical power relations.

A central finding is that the relationship between coaches and physiotherapists oscillates between working alongside, working together, and working against each other. These constellations should not be understood as fixed types of collaboration, but as situational modes of intersystemic interaction. Alongside constellations become visible where both professional groups perform their tasks in parallel, with limited systematic coordination and only occasional exchange. Together constellations emerge where spatial proximity, personal trust, reciprocal understanding, and case-related communication enable joint problem-solving. Against constellations occur when health- and performance-related expectations are mobilised against one another, for example in return-to-sport decisions, load management, competition readiness, information disclosure and role boundary negotiations. With regard to the aim of this study, this spectrum shows that systemic collaboration and intersystem sensitivity are not fixed characteristics of elite sport support teams, but situationally produced and structurally conditioned modes of interaction between sport and health.

The findings reveal both similarities and differences in the self-descriptions of coaches and physiotherapists. Both groups describe themselves as relevant and necessary parts of elite sport support teams. Both emphasise responsibility, high availability and the practical importance of their work for athletes. Both are also confronted with the tension between short-term performance expectations and longer-term athlete health. However, their role realities differ considerably. Physiotherapists describe an expanded and partly diffuse role that includes therapeutic, emotional, logistical, and availability-related tasks, often under precarious or insufficiently institutionalised working conditions. Coaches, by contrast, describe a more clearly delimited and hierarchically central role focused on performance development, team coordination, and decision-making authority. This asymmetry is not incidental. It forms a structural condition of interaction between both professional groups.

The reciprocal descriptions of the other professional domain further illustrate this intersystemic structure. Physiotherapists generally recognise the coaches’ authority in sport-specific training and competition preparation. At the same time, they tend to describe coaches as performance-driven, risk-accepting, and sometimes insufficiently sensitive to medical-therapeutic complexity. Coaches, in turn, recognise physiotherapists as indispensable for physical care, prevention, rehabilitation, and competition readiness. However, they often interpret physiotherapy primarily through the lens of performance functionality: Physiotherapists are expected to restore, maintain, or optimise the athlete’s body for training and competition. They are criticised when they appear too cautious, too academically oriented, insufficiently available, or too dominant in relation to training decisions. Thus, both groups acknowledge the relevance of the other domain, but they do so from the standpoint of their own systemic reference.

This reciprocal observation confirms and extends previous research on the culture of risk in elite sport. Pain, injury, and health impairments are not external disturbances of elite sport, but often become normalised features of performance production (Nixon, 1992, 1994; Safai, 2003; Curry and Strauss, 1994). Decisions about treatment duration, return to sport, playing hurt, and presenteeism therefore become practical sites where sport and health logics intersect (Theberge, 2007; Malcolm, 2006; Mayer and Thiel, 2011; Mayer et al., 2020). Our findings show that these tensions are not merely ethical dilemmas at the level of individual decision-making. They are also intersystemic coordination problems. The same situation can be observed differently depending on whether it is primarily framed as a performance problem, a health problem, an organisational problem, or a professional autonomy problem.

This is particularly evident in return-to-sport and load management decisions. Physiotherapists describe these situations as conflict-prone because they require balancing medical-therapeutic judgement against performance pressure and competition relevance. Coaches, by contrast, often expect physiotherapists to contribute to functional readiness and to provide information that supports training and competition decisions. In such situations, conflict potentials arise not because one side necessarily acts irrationally, but because both sides act plausibly within different functional orientations. From the perspective of elite sport, the timely restoration of performance capacity is highly relevant. From the perspective of health, the prevention of further injury and the protection of the athlete as patient are central. Intersystem sensitivity becomes necessary precisely because both logics are legitimate, but not automatically compatible.

The findings also indicate that collaboration between coaches and physiotherapists is not only a knowledge problem. It is also a system and structure problem. Certainly, mutual knowledge deficits play a role. Physiotherapists expect coaches to better understand physiological processes, wound healing, and medical-therapeutic complexity. Coaches expect physiotherapists to better understand sport-specific loads, competitive demands, and the practical urgency of elite sport. However, knowledge alone cannot solve the observed problems if collaboration remains structurally underdetermined. In many contexts, communication is informal, person-dependent, and situational rather than institutionally secured. Physiotherapists often work under limited availability, low remuneration, or freelance conditions, while coaches are more consistently embedded in clubs, federations, and training structures. This creates unequal action capacities and reinforces asymmetrical expectations.

Against this background, intersystem sensitivity should not be understood as a purely individual attitude or communicative skill. It is a structural professional competence. At the personal level, it involves the capacity to recognise the system reference of one’s own professional practice, to understand the legitimacy of the other domain, and to anticipate how divergent definitions of success may generate conflict potentials. At the structural level, however, intersystem sensitivity requires organisational arrangements that make such reflexivity practically possible. Without time, proximity, role clarity, and institutionalised communication formats, even highly sensitive professionals may remain unable to coordinate their actions effectively.

From this perspective, the empirical findings can be differentiated across three interconnected analytical dimensions (Luhmann, 1975). At the societal level, the functional differentiation of sport and health generates divergent criteria of relevance and success. At the organisational level, clubs, federations, and training centres translate these references into decision premises, role assignments, contractual arrangements, resource allocations, and procedures for handling information. At the interactional level, these organisational arrangements and societal references become effective in concrete case discussions, for example concerning return-to-sport, load management, or competition readiness. Organisational multireferentiality thus does not automatically result in a balanced integration of the different functional perspectives. Organisations may prioritise sporting performance in their decision structures, while the consideration of health-related expectations remains dependent on local arrangements, available resources, and the organisational position of physiotherapists. This helps to explain why intersystem sensitivity requires not only individual perspective-taking, but also organisational structures that allow divergent professional logics to be articulated, translated, and coordinated without eliminating their differences.

This has implications for professionalisation in both fields. Professionalisation does not automatically lead to better collaboration. On the contrary, increasing professional autonomy, stronger occupational identities, and more differentiated knowledge bases may initially increase expectation uncertainty between professional groups. Physiotherapists who claim broader expertise in movement observation, prevention, and training-related assessment may challenge coaches’ traditional decision-making authority. Coaches who understand themselves as central coordinators of the support team may interpret such claims as interference. Bilateral professionalisation therefore requires the re-coordination of relationships under conditions of increased autonomy. In this sense, systemic collaboration and intersystem sensitivity are not optional additions to professional work in elite sport. They are necessary competences for handling the consequences of professionalisation itself.

Practical optimisation should therefore address both behaviour and structures. At the behavioural level, training programmes could promote intersystemic perspective-taking, basic understanding of the other professional domain, and reflexive awareness of explicit and implicit power relations. Coaches would benefit from a stronger understanding of medical-therapeutic processes, confidentiality issues, and the temporal logic of healing. Physiotherapists would benefit from a stronger understanding of training logic, competition pressure, and the performance-related temporality of elite sport. Interdisciplinary and case-based continuing education could be particularly useful here, because it allows both groups to work through realistic scenarios rather than abstract role descriptions (Thiele and Schierz, 2006). At the structural level, elite sport organisations should create more reliable conditions for systemic collaboration. This includes clear role and decision architectures, negotiated procedures for return-to-sport decisions, regular and institutionally secured communication formats, and transparent rules for handling athlete-related health information. A shared working language is also needed, not in the sense of dissolving professional differences, but as a symbolic structure that allows different professional perspectives to become mutually understandable and practically connectable. Such arrangements would reduce the dependence of collaboration on personal trust, local proximity, and *ad hoc* improvisation. At a broader policy level, these organisational conditions are themselves shaped by national arrangements for funding, professional education, employment, and support provision. However, the present data do not permit a systematic assessment of such policy frameworks or their implementation.

Methodologically, the study demonstrates the potential of using sociological systems theory as a heuristic framework for empirical research (e.g., Nassehi, 1998, 2008; Vogd, 2007), while at the same time integrating a comparatively rare emphasis on the level of society, rather than focusing exclusively on organizations or interactions (Henkel, 2010). Systems theory does not provide a simple variable model that can be directly operationalised in a linear way. Rather, it offers a conceptual vocabulary for observing how communication, expectations, roles, and system references structure social situations. In this study, systems theory sensitised the analysis to distinctions between sport and health, to conflict potentials, and to the contingency of collaboration. The empirical contribution therefore does not consist in testing systems theory in a narrow sense, but in using it as an observational framework for reconstructing how intersystemic expectations become visible in professional self-descriptions, reciprocal role interpretations, and descriptions of cooperation.

Also, several limitations must be considered. First, the findings represent the perspectives of the interviewed coaches and physiotherapists and cannot be generalised to German elite sport as a whole. The sample covers a broad range of Olympic sports, but the structural conditions of collaboration differ across sports, clubs, federations and performance levels as well as between genders: The organisational models of physiotherapy provision varied considerably, ranging from on-site physiotherapy treatment rooms and staff at training grounds, to athletes receiving treatments on demand from privately funded physiotherapists, and to clubs/federations relying on collaborations with external private physiotherapy practices as needed. Accordingly, the collaboration differed substantially across these models, particularly with respect to the availability and continuity of physiotherapy treatments. Second, the data are based on retrospective self-reports and are therefore shaped by subjective reconstruction, selective memory and possible social desirability effects (Flick et al., 2010). A related limitation concerns the fact that, due to the COVID-19 pandemic, the data had to be collected via telephone interviews. As the interviewers and interviewed coaches and physiotherapists were unfamiliar with one another prior to the interviews, the interactional dynamics may have differed from those of face-to-face interviews. Consequently, the telephone-based format may have limited the development of rapport and the depth of interaction, making it possible that additional nuances would have emerged in face-to-face interviews but remained unexplored in the telephone interviews (Gläser and Laudel, 2010). Third, the study focuses primarily on coaches and physiotherapists. Other relevant actors, especially athletes but also physicians, psychologists, sport scientists, managers and other officials, were not systematically included in the present analysis. Their perspectives would be necessary to reconstruct the wider support team ecology more comprehensively. Fourth, the analytical focus on conflict potentials and optimisation needs may underrepresent already successful practices of collaboration. Future studies should therefore examine not only where intersystemic collaboration fails, but also where and how it is already stabilised in effective routines.

Finally, the concept of intersystem sensitivity itself requires further theoretical and empirical development. While the present study shows that the concept is useful for analysing collaboration between coaches and physiotherapists, its practical implementation and effects remain open questions. Future research should investigate how intersystem sensitivity can be developed through education and organisational design, how it can be observed in everyday interaction, and whether it contributes to more stable collaboration, better athlete care and more sustainable performance development. In this respect, the study provides a conceptual and empirical basis for further research on professional collaboration in functionally differentiated elite sport environments.

## 6 Conclusion

The analysis presented in this article shows that collaboration between coaches and physiotherapists in elite sport support teams can be further understood as an intersystemic relationship situated between sport and health. This relationship is not simply characterised by cooperation or conflict, but by a situational oscillation between working alongside, working together, and working against each other. These modes of interaction reflect different ways of managing tensions between performance-oriented and health-oriented expectations. The study demonstrates that these tensions are rooted not only in individual misunderstandings or professional differences, but also in divergent definitions of success, asymmetrical role expectations, organisational conditions, and communication structures. By conceptualizing collaboration in elite sport support teams as an intersystemic constellation between sport and health, this article contributes to a more differentiated understanding of multiprofessional support work. It shows that effective professional collaboration depends not only on expertise, but also on the capacity to manage functionally distinct societal expectations.

## Data Availability

The datasets presented in this article are not readily available because restrictions apply to their availability, as they were used under ethical permission for the current study. Due to the sensitivity of the data, access is restricted to ensure compliance with data protection regulations and to safeguard participants’ privacy. Requests to access the datasets should be directed to a.steinmann@dshs-koeln.de.

## Footnotes

1 A concise example of this can be found in Nassehi (2010, pp. 12ff.), who provides a pointed sociological observation of the intersystem (de)sensitivity of different social actors within the ethics committee of a large hospital (e.g., physicians, chaplains, hospital administration, a legal expert, representatives of nursing staff, and a patient advocate).

2 Science of society, for example, observes the world according to the code true/false, thereby structuring all operations in relation to the distinction between truth and non-truth. The education system, by contrast, stabilizes its operations through career-shaped selection processes, in which individual educational trajectories are generated, differentiated, and allocated. The political system processes communication through the medium of power, with the function of producing collectively binding decisions that can be enforced within its societal environment. The economy operates along the binary code payment/non-payment and addresses the problem of scarcity by ensuring the provision of present and future needs via the medium of monetary payments (Luhmann, 1997).

3 For example, teachers in schools primarily orient the design of instruction and assessments to the social attainability of their students’ achievements; physicians are professionally engaged in maintaining or restoring the health of their patients; members of the legal profession aim, without question, for a just legal assessment of their clients (Luhmann, 1997).

4 This solution is by no means the only option. For the established approach of resolving structural social conflicts institutionally through asymmetrical decision-making structures and hierarchical regulatory mechanisms see Simon (2022, pp. 78ff.).
