# Supplementary material for: Together, alongside or against each other? A qualitative content analysis on the intersystem sensitivity of coaches and physiotherapists in German elite sport support teams
Source: Front Sociol. 2026 Aug 19;11:1870432. doi: 10.3389/fsoc.2026.1870432 (PMC13533824; doi:10.3389/fsoc.2026.1870432)
Supplement: Supplementary file 1 [file Data_Sheet_1.pdf]

Supplementary Material

Supplementary Table 1. Overview of the category system (including codes).

**Oberkategorie (Codes)**	**Unterkategorie (Codes)**	**Definition**	**Zusätzliche Kodier- und Abgrenzungsregeln**	**Ankerbeispiel**
I. Beschreibung des eigenen beruflichen Handlungsfelds (905)	I.1 Berufsspezifische Aufgaben und Qualifikation (411)	Aufgaben, Tätigkeiten und Entscheidungen die explizit in den eigenen Verantwortungs- und Entscheidungsbereich fallen und nicht in den des anderen beruflichen Handlungsfelds. Für Physiotherapeut*innen sind das medizinische Aufgaben, z. B. präventiver oder rehabilitativer Natur. Für Trainer:innen bedeutet das, sportartspezifische, trainingswissenschaftliche Aufgaben, wie Trainingsgestaltung, Trainingsplanung etc.	Mit Entscheidungen meinen wir hier solche, für die eine Person die Entscheidungshoheit hat und nicht auf einen Konsens mit anderen angewiesen ist. Wir kodieren hier auch Wege zur Berufszulassung, Ausbildungen und Fortbildungen. Dazu zählt auch (eigeninitiativ) angeeignetes Fachwissen.	„Also durchschnittlicher Arbeitstag sieht so aus, dass ich morgens früh Training habe, individuell mit den Sportlern nach Trainingsplan und dann dazwischen, klar, das Übliche: Trainingsplanung, Trainingsdokumentation, Treffen mit irgendwelchen Leuten vom Verband oder von der Nationalmannschaft, dann abends Training, ja so sieht mein Tag aus. Abends, wenn ich Glück habe, habe ich nach dem Training Feierabend. Wenn ich Pech habe, muss ich noch Trainingsplanung machen oder irgendwelche anderen Geschichten." (#T12)
	I.2 Nicht berufsspezifische Aufgaben (15)	Berufliche unpezifische Aufgaben, die in der Rolle des eigenen beruflichen Alltags übernommen werden. Diese könnten auch von anderen Personen im Unterstützungsteam wahrgenommen warden		„Also, es kann auch sein, dass, ich sage mal, ein Tape auf den Schuh noch mal mit darauf klebe oder mich darum kümmere, dass die Flaschen vielleicht mit rüber getragen werden." (#P6)

	I.3 Strukturelle Gegebenheiten (249)	Aussagen zur Organisation, zum Aufbau, zur Verfügbarkeit und zur Infrastruktur des eigenen Berufsfeldes. Aussagen zur Finanzierung und Bereitstellung der Strukturen. Für Trainer*innen sind das Strukturen, die das Training, den Verband und Vereine betreffen. Für Physiotherapeut*innen sind das Strukturen, die die medizinische Infrastruktur und das therapeutische Versorgungssystem betreffen		„Also, die Athleten in der Olympia-Quali spielen 26 Turniere und kein Trainer betreut 26 Turniere (I: Ok.) Also, wir haben auch ein wechselndes Trainerteam vor Ort.“ (#T4)
	I.4 Anstellungsverhältnis und Vergütung (36)	Vertragliche Grundlage und Bezahlung der spezifischen Personen		„Ich bin als Trainer am Olympiastützpunkt angestellt.“ (#T5)
	I.5 Systemkonflikt (128)	Konflikte und Dilemmata zwischen Gesundheits- und Leistungsorientierung aus Sicht des eigenen beruflichen Handlungsfeldes.	Es kann der (empfundene) vorhandene Konflikt sein oder eine Situation, die Potenzial für einen Systemkonflikt beinhaltet.	„Also, ich stelle jetzt Gesundheit, genauso wie der Athlet, einfach nur als eine Art Basis oder Voraussetzung halt. Das Ziel ist eigentlich immer Zeiten, Weiten und Medaillen.“ (#P11)
	I.6 Beziehung zu den Athlet*innen (66)	Wechselseitiges Verhältnis zu den betreuten Athlet*innen		„Da ist inzwischen ein intensives, kameradschaftliches Verhältnis entstanden, man tauscht sich aus […] in Richtung Freundschaft oder gar Kameradschaft.“ (#P1)

II. Beschreibung des anderen beruflichen Handlungsfelds (473)	II.1 Berufsspezifische Aufgaben und Qualifikation (200)	Aufgaben, Tätigkeiten und Entscheidungen die explizit in den eigenen Verantwortungs- und Entscheidungsbereich fallen und nicht in den des anderen beruflichen Handlungsfelds. Für Physiotherapeut*innen sind das medizinische Aufgaben, z. B. präventiver oder rehabilitativer Natur. Für Trainer:innen bedeutet das, sportartspezifische, trainingswissenschaftliche Aufgaben, wie Trainingsgestaltung, Trainingsplanung etc.	Mit Entscheidungen meinen wir hier solche, für die eine Person die Entscheidungshoheit hat und nicht auf einen Konsens mit anderen angewiesen ist. Wir kodieren hier auch Wege zur Berufszulassung, Ausbildungen und Fortbildungen. Dazu zählt auch (eigeninitiativ) angeeignetes Fachwissen.	„Die Hauptaufgaben der Trainerinnen sind natürlich die Weiterentwicklung auf sportlicher Ebene der Athleten." (#P7)
	II.2 Nicht berufsspezifische Aufgaben (12)	Beruflich unpezifische Aufgaben, die in der Rolle des eigenen beruflichen Alltags übernommen werden. Diese könnten auch von anderen Personen im Unterstützungsteam wahrgenommen werden		„Also ich finde, ein Physio ist auch immer so ein GUTE-Laune-Bär. Der immer dafür sorgt, dass die Laune gut hochgehalten wird, falls der Trainer mal wieder einen schlechten Tag hatte." (#T5)
	II.3 Strukturelle Gegebenheiten (138)	Aussagen zur Organisation, zum Aufbau, zur Verfügbarkeit und zur Infrastruktur des eigenen Berufsfeldes. Aussagen zur Finanzierung und Bereitstellung der Strukturen. Für Trainer*innen sind das Strukturen, die das Training, den Verband und Vereine betreffen. Für Physiotherapeut*innen sind das		„Also, wir haben jetzt eigentlich so gesagt, dass die Bundeskader zweimal in der Woche eine Physiotherapie wahrnehmen sollten (I: Ja) wenn sie es in ihr zeitliches Budget noch reinbringen, was manchmal gar nicht so einfach ist, weil grad die Schüler da sehr, sehr viel zeitliche Belastung haben." (#T2)

		Strukturen, die die medizinische Infrastruktur und das therapeutische Versorgungssystem betreffen		
	II.4 Anstellungsverhältnis und Vergütung (39)	Vertragliche Grundlage und Bezahlung der spezifischen Personen		„Die Trainer, boah das ist auch/ es gibt definitiv bei uns auch einige hauptamtliche Trainer, da sind auch Bundestrainer dabei und es gibt andere Trainer, die das nebenberuflich beziehungsweise auf halber/ Noch einen anderen Beruf ausüben und nicht nur den reinen Trainerjob.“ (#P19)
	II.5 Systemkonflikt (33)	Konflikte und Dilemmata zwischen Gesundheits- und Leistungsorientierung aus Sicht des anderen beruflichen Handlungsfeldes.	Es kann der (empfundene) vorhandene Konflikt sein oder eine Situation, die Potenzial für einen Systemkonflikt beinhaltet.	„Also, (lacht) ja, klar, manchmal ist es so, dass man einen wirklich schnell wieder hinkriegt, dass er zumindest den wichtigen Wettkampf bestreiten kann oder dass man hingeht und sagt, ‚wir müssen doch mal ein bisschen länger uns damit beschäftigen über Wochen hinweg‘ um zu schauen, ob der Athlet seine Probleme in den Griff kriegt und was wir vielleicht auch im Training anders machen müssen, dauerhaft, um die und die Überlastungsschäden vermeiden zu können. Also, da gibt es unterschiedliche Zielsetzungen.“ (#T8)
	II.6 Beziehung zu den Athlet*innen (51)	Wechselseitiges Verhältnis zu den betreuten Athlet*innen		„Also, bei uns in der Physiotherapie ist, ein ganz ganz großes Vertrauenspotential da. Uns erzählt der Athlet sicherlich Dinge, die er dem Trainer nicht unbedingt erzählt.“ (#P24)

III. Kooperations-beschreibung (710)	III.1 Kooperation Trainer*in und Physiotherapeut*in (252)	Beschreibung der Zusammenarbeit zwischen Trainer*innen und Sportphysiotherapeut*innen.	Beinhaltet alltägliche Interaktionen und Absprachen zwischen Trainer*innen und Physiotherapeut*innen.<br><br>Die Kategorie betrifft nicht individuell fachliche Aufgabenbeschreibungen, individuell fachliche Aufgabenbeschreibungen sind den Kategorien „Beschreibung des eigenen / anderen beruflichen Handlungsfeldes“ zuzuordnen	„Dann gibt es natürlich Absprachen über den mentalen Bereich oder den psychologischen Bereich.“ (#T1)
	III.2 Kooperation Physiotherapeut*innen, Trainer*innen und Dritte (195)	Beschreibungen der Zusammenarbeit zwischen Physiotherapeut*in und Trainer*in plus ein oder mehrere Partner*innen	Es müssen nicht alle drei (oder mehr) Parteien direkt miteinander in Kontakt sein.	„Dann ist, am Wettkampftag immer ein Mannschaftsarzt mit vor Ort, plus mindestens ein Sportphysiotherapeut und dann muss dort sofort reagiert werden und dann ist der erste Weg natürlich erstmal ab zum Arzt. [...] also ohne gescheite Diagnostik GEHT es definitiv nicht.“ (#P23)
	III.3 Strukturelle Gegebenheiten (60)	Aussagen zur Organisation der Interaktion, Kommunikation und des Austauschs zwischen Physiotherapeut*innen und Trainer:innen.	Es geht nicht um die Verfügbarkeit von Physiotherapeut*innen für die Behandlung von Athlet*innen.	„Bei uns im Team ist es so, der Physio hat seine Hauptarbeitszeit, wenn der Tag geschafft ist.“ (#T3)
	III.4 Kritik und Konflikte (134)	Kritik und Konflikte, die in der Zusammenarbeit auftreten. Muss nicht persönlicher Natur sein, sondern kann auch organisatorische Konflikte beinhalten. Kritik birgt Konfliktpotenzial und kann,	Hier werden keine Konflikte kodiert, die zwischen dem System Leistungssport und dem Medizinsystem auftreten.	„Also, Konfliktpotential sicherlich, wenn der Informationsfluss nicht so ist, wie er sein sollte.“ (#T13)

		muss aber nicht, den betreffenden Personen gegenüber geäußert werden.		
	III.5 Hierarchie (69)	Beschreibungen von Über- oder Unterordnungen in der Zusammenarbeit (z. B. mehr oder weniger strenge Rangordnung in der Kooperation)	Werden aus der Hierarchie explizite Konflikte benannt, dann werden diese in der Unterkategorie „Kritik und Konflikte“ kodiert.	„Ich habe den Hut auf.“ (#T2)
IV. Optimierungsansätze (260)	IV.1 Fachlich im eigenen beruflichen Handlungsfeld (34)	Berufsspezifische Verbesserungsvorschläge und Idealvorstellungen des eigenen beruflichen Handlungsfelds, die auf eine Veränderung abzielen.	Betrifft Verbesserungsvorschläge und Idealvorstellungen in Zusammenhang mit der Kategorie „Berufsspezifische Aufgaben und Qualifikation“. Wir kodieren hier auch Kritik, die darauf hinweist, dass aktuelle Zustände nicht optimal sind, ohne konkrete Verbesserungsvorschläge zu nennen.	„Ja, schwierig. Ich glaube, dass die / dass das, was man sich MAL überlegt hat in Deutschland als Ausbildungssystem für Sportphysiotherapeuten, das ist ja eben die DOSB-Lizenz. Dass da sicherlich ein großer Reformbedarf ist. Ja, weil eben sich alles weiterentwickelt. (Lacht) [...] Da ist sicherlich viel, viel Bedarf und auch sicherlich, aus meiner Sicht MEHR Ausbildungszeit erforderlich.“ (#P2)
	IV.2 Fachlich im anderen beruflichen Handlungsfeld (22)	Berufsspezifische Verbesserungsvorschläge und Idealvorstellungen in anderen beruflichen Handlungsfeldern, die auf eine Veränderung abzielen. Andere berufliche Handlungsfelder können sowohl Physiotherapeut*innen oder Trainer*innen als auch Dritte beinhalten	Betrifft Verbesserungsvorschläge und Idealvorstellungen in Zusammenhang mit der Kategorie „Berufsspezifische Aufgaben und Qualifikation“. Wir kodieren auch Kritik, die darauf hinweist, dass aktuelle Zustände nicht optimal sind, ohne konkrete Verbesserungsvorschläge zu nennen.	„Der muss Bewegungen beobachten können. Davon sind wir leider in Deutschland, in der täglichen Realität von Olympiastützpunkten zu weit entfernt. Da sollte mehr drauf hingewirkt werden, dass der Physiotherapeut einfach auch ein Trainer ist. Der eben bestimmte, sehr körperbezogene Aufgaben hat.“ (#T7)

	IV.3 Kooperation (57)	Verbesserungsvorschläge und Idealvorstellungen die die Zusammenarbeit betreffen und auf eine Veränderung abzielen.	Wir kodieren auch Kritik, die darauf hinweist, dass aktuelle Zustände nicht optimal sind, ohne konkrete Verbesserungsvorschläge zu nennen.	„Naja. Auf jeden Fall in der Kommunikation. (lacht) Dass es mal eine engere Absprache gibt zwischen Trainer und medizinischer Abteilung. Dass man einfach auch feinjustieren kann. Ich würde mir zum Beispiel auch sehr wünschen auch beim Training mit dabei zu sein, um einfach Bewegungsabläufe bei den Spielerinnen zu sehen. Und teilweise auch die Wertigkeit. Wir hatten jetzt neulich die Situation, dass eine Spielzeit verändert wurde. Also nach vorne verlegt wurde. Und alle wurden gefragt, außer die Physiotherapeuten, ob das zeitlich überhaupt möglich wäre.“ (#P6)
	IV.4 Strukturelle Gegebenheiten (147)	Strukturelle Verbesserungsvorschläge und Idealvorstellungen, die auf eine Veränderung abzielen.	Wir kodieren auch Kritik, die darauf hinweist, dass aktuelle Zustände nicht optimal sind, ohne konkrete Verbesserungsvorschläge zu nennen.	„Ich weiß, dass die Physiotherapeuten haben ja auch eine Forderung an den DOSB mit der Bitte: Die und die Bereiche sind aus physiotherapeutischer Sicht zu optimieren und ein Kerngebiet dessen war halt einfach/ da ging es auch viel um organisatorische Geschichten, wie dass doch faire Honorierungen stattfinden, dass doch Speis und Unterkunft und all das bezahlt werden. Wo ich dachte, das hab ich in meinem Bereich. Bis hin zu, dass ordentliche Arbeitsbedingungen geschaffen werden und ordentliche Arbeitszeiten.“ (#T3)
